# The Cardioprotective Effect of Vitamin E (Alpha-Tocopherol) Is Strongly Related to Age and Gender in Mice

**DOI:** 10.1371/journal.pone.0137405

**Published:** 2015-09-02

**Authors:** Xiao-Xia Hu, Li Fu, Yan Li, Ze-Bang Lin, Xiang Liu, Jing-Feng Wang, Yang-Xin Chen, Zhi-Ping Wang, Xi Zhang, Zhi-Jun Ou, Jing-Song Ou

**Affiliations:** 1 Division of Cardiac Surgery, The First Affiliated Hospital, Sun Yat-sen University, Guangzhou, 510080, P.R. China; 2 The Key Laboratory of Assisted Circulation, Ministry of Health, The First sAffiliated Hospital, Sun Yat-sen University, Guangzhou, 510080, P.R. China; 3 Guangdong Province Engineering Laboratory for Diagnosis and Treatment of Vascular Diseases, The First Affiliated Hospital, Sun Yat-sen University, Guangzhou, 510080, P.R. China; 4 Division of Hypertension and Vascular Diseases, The First Affiliated Hospital, Sun Yat-sen University, Guangzhou, 510080, P.R. China; 5 Department of Cardiology, Sun Yat-sen Memorial Hospital, Sun Yat-sen University, Guangzhou, 510120, P.R. China; 6 Guangdong Province Key Laboratory of Arrhythmia and Electrophysiology, Sun Yat-sen Memorial Hospital, Sun Yat-sen University, Guangzhou, 510120, P.R. China; Indiana University School of Medicine, UNITED STATES

## Abstract

Vitamin E (VitE) only prevented cardiovascular diseases in some patients and the mechanisms remain unknown. VitE levels can be affected by aging and gender. We hypothesize that age and gender can influence VitE’s cardioprotective effect. Mice were divided into 4 groups according to age and gender, and each group of mice were divided into a control group and a VitE group. The mice were administered water or VitE for 21 days; Afterward, the cardiac function and myocardial infarct size and cardiomyocyte apoptosis were measured after myocardial ischemia reperfusion(MI/R). VitE may significantly improved cardiac function in young male mice and aged female mice by enhancing ERK1/2 activity and reducing JNK activity. Enhanced expression of HSP90 and Bcl-2 were also seen in young male mice. No changes in cardiac function and cardiac proteins were detected in aged male mice and VitE was even liked to exert a reverse effect in cardiac function in young mice by enhancing JNK activity and reducing Bcl-2 expression. Those effects were in accordance with the changes of myocardial infarction size and cardiomyocyte apoptosis in each group of mice. VitE may reduce MI/R injury by inhibiting cardiomyocyte apoptosis in young male mice and aged female mice but not in aged male mice. VitE was possibly harmful for young female mice, shown as increased cardiomyocyte apoptosis after MI/R. Thus, we speculated that the efficacy of VitE in cardiac protection was associated with age and gender.

## Background

Vitamin E (VitE), a lipophilic vitamin, has a strong antioxidative effect and is reported to be effective for the primary and secondary prevention of cardiovascular(CV) diseases [1–3]. Epidemiologic data showed that the incidence rate of CV events decreased due to diets rich in VitE [4,5]. These effective observations represent the foundation for further clinical trials. However, there were some controversial results, which indicated that VitE was ineffective in decreasing either CV events or mortality[6–9]. On the basis of these results, a new viewpoint was proposed in which VitE may only be effective in specific patients suffering from CV problems [10]. VitE was demonstrated to have protective efficacy in patients who have both CV disease and type 2 diabetes mellitus. However, there may be other unknown factors that can influence the effect of VitE on CV diseases.

The serum and visceral levels of CoQ and α-tocopherol (α-toco) decreased during aging in rats [11], which indicated that age may modulate the effect of VitE. Moreover, two subsequent clinical trials also indicated that age could affect the protective efficacy of VitE for CV diseases [1,12]. The Women’s Health Study showed that when 600 IU/d natural α-toco was administered, the incidence of CV diseases, especially cardiac death, decreased. Further study showed that VitE could significantly reduce the incidence of adverse CV events (stroke excluded) in a group of patients aged over 65 [1]. Rodrigo et al. reported that a combined supplementation of omega-3 polyunsaturated fatty acids (PUFAs) with vitamins C and E could significantly reduce the incidence of atrial fibrillation in aged patients (over 60 years old) undergoing cardiac surgery. However, in young patients (younger than 60), this treatment showed no marked effect [12]. The results of these studies indicate that age may influence the efficacy of VitE protection against CV diseases.

Animal models showed that CV protective efficacy may be closely related to gender. In myocardial ischemia-reperfusion (MI/R) injury models of young female rats, VitE was effective in reducing oxidative stress; however, no changes in cardiac performance were found during this procedure [13,14]. In models using young male animals, VitE not only reduced oxidative stress but also improved cardiac function [15,16]. Another study showed that compared with the hearts of males, female hearts were better able to endure ischemia-reperfusion injury. Most likely, this change occurred because the α-toco level decreased more dramatically in males than in females. The α-toco level of the normal female animals was also higher than that of the males [17]. The above findings in animal models indicate that gender can affect the protective efficacy of VitE.

On the basis of the results of the research described above, we hypothesize that age and gender are influential factors in the protective effect of VitE during CV events. The aim of this study is to investigate the role of age and gender in the protective efficacy of VitE in animal models.

## Methods

### Reagents and antibodies

Vitamin E (VitE, (+)-α-tocopherol acetate, α-toco) was purchased from Sigma-Aldrich (St. Louis, MO, USA). Primary antibodies, such as JNK, p-JNK, ERK1/2 and p-ERK1/2, were from Cell Signaling Technology (Beverly, MA, USA). Anti-rabbit secondary antibody and anti-GAPDH primary antibody were purchased from ProteinTech Group, Inc. (Chicago, IL, USA), and ECL reagents and anti-HSP90 primary antibody were obtained from Santa Cruz Biotechnology (California, USA).

### Animal pretreatment

The experimental protocol was approved by the Animal Ethics Commission of the First Affiliated Hospital, Sun Yat-sen University. The animal work was performed in accordance with the Guiding Principles for the Care and Use of Animals based on the Declaration of Helsinki. The Experimental Animal Center of Sun Yat-sen University provided 3- and 12-month-old C57BL/6J mice, which were housed in a controlled environment with a temperature of 25°C and a 12 h light/dark cycle. During the study period, food and water were provided ad libitum. The mice were divided into 4 groups according to gender and age: young females (3 months old), young males (3 months old), aged females (12 months old) and aged males (12 months old). The mice in each group were randomly divided into 2 subgroups, the α-toco group and the control group. The number of mice used in each subgroups as well as the body weight were measured and recorded. As α-toco is the most abundant form of Vit E in plasma and is most biologically active [10], α-toco was used for substitution of VitE in this study. The mice divided into α-toco group were administered with α-toco (dissolved/suspended in sterile water, 100 IU/kg) via oral gavage for 3 weeks. Sterile water was administered in the same manner to the control group.

### MI/R protocol

The anesthesia, intubation and ligation of the left anterior descending coronary (LADC) were performed as previously described [18]. Briefly, mice were fully anesthetized with sodium pentobarbital (50 mg/kg) and intubated and connected to a rodent ventilator. The mice were positioned on their right sides, a left thoracotomy was performed at the fourth intercostal, and the chest cavity was opened to expose the heart. At approximately 1–2 mm from the tip of the left atrium, a slipknot was made on the LADC using an 8.0 nylon suture with a small piece of polyethylene-10 tube underneath. A purse-string suture was placed around the incision and was tied to close the chest immediately after the lung was inflated. After a 45-minute ischemic procedure, the chest was reopened by loosening the purse-string suture. The heart was reperfused by loosening the nylon suture. Approximately 24 hours after the surgical procedure, the cardiac functional parameters were recorded for the mice from survived MI/R procedure. After that, myocardial infarct size, cardiomyocyte apoptosis and the expression and phosphorylation of cardiac proteins were determined.

### Measurement of cardiac function

Approximately 24 hours after the surgical procedure, two-dimensional transthoracic echocardiography was performed on to obtain data regarding cardiac structure and function, including the intraventricular septal end-diastolic dimension (IVSd), the LV internal dimension at end diastole (LVIDd), the LV internal dimension at end systole (LVISd), the LV ejection fraction (EF; shown as a %) and the LV fractional shortening (FS; shown as a percentage), which were calculated from M-mode images. During the procedure, mice were under anesthesia with isoflurane supplemented with 100% O2. Transthoracic echocardiography was performed to obtain B-mode and M-mode images using a 30-MHz probe connected to a Vevo 2100 (Visualson-cs) imaging system.

### Determination of myocardial infarct size

Approximately 24 hours after the surgical procedure, the mice were anesthetized using pentobarbital and administered heparin (1 U/g, IV). After LADC reocclusion, approximately 1 ml 5% Evans blue dye dissolved in PBS was injected into the aortic root. The heart was then excised and frozen at -80°C. The heart was transversely cut into 5–6 slices and incubated in 1% 2,3,5-triphenyltetrazolium chloride (TTC, Sigma Aldrich) solution (pH = 7.34, T = 37°C) for 15 minutes. The heart slices were then fixed in 10% formalin overnight. The left ventricular tissue became red (TTC, ischemic risk region) or blue (Evans blue, nonrisk region), and the infarcted tissue was pale. The myocardial infarct size was determined as the percentage of the risk region.

### TUNEL assay for the assessment of apoptotic cell death

Cardiomyocyte apoptosis was assessed using terminal deoxy-nucleotidyl transferase-mediated dUTP-biotin nick end-labeling reaction (TUNEL) labeling of the 3′-OH ends of DNA in paraffin-embedded heart sections 24 h after MI/R. The hearts were harvested from MI/R mice, fixed in 10% formalin, embedded in paraffin, and sectioned at 6 μm. The sections were incubated for 1 h at 65°C, deparaffinized in xylene and rehydrated using a graded series of ethanol (absolute, 95%, 80% and 70%). Then, the TUNEL experiment was performed according to the manufacturer's instructions. The staining was viewed under a light microscope.

### Immunoblotting (IB)

Approximately 24 hours after the surgical procedure, the mice hearts were isolated. The heart tissues were homogenized in pH 7.4 ice-cold lysis buffer containing 1% protease and phosphatase inhibitors, 20 mM Tris-HCl, 1% sodium deoxycholate, 10 mM NaF, 1% Triton X-100, 2.5 mM EDTA and 0.1% SDS. The lysates were sonicated and then centrifuged at 11,000 g for 30 min. The supernatant was transferred to a 1.5-ml tube. The protein concentration was quantitated using the BSA method. Then, the IB process was conducted as previously described [19]. Antibodies for detecting HSP90, JNK, ERK1/2, GAPDH, p-JNK p-ERK1/2 and Bcl-2 were used. The primary antibody binding was detected using horseradish peroxidase-conjugated secondary antibody for 1 h at room temperature and visualized using the ECL method.

### Statistical analysis

Data are presented as means ± SD unless specially indicated. Statistical evaluations were performed using unpaired student t tests for 2 independent samples. Nonparametric test was used for comparing infarct size. A p value of less than 0.05 was deemed statistically significant. All statistical analyses were performed using SPSS 17.0.

## Results

### Effects of α-toco on left ventricular function after MI/R

**Fig 1:**
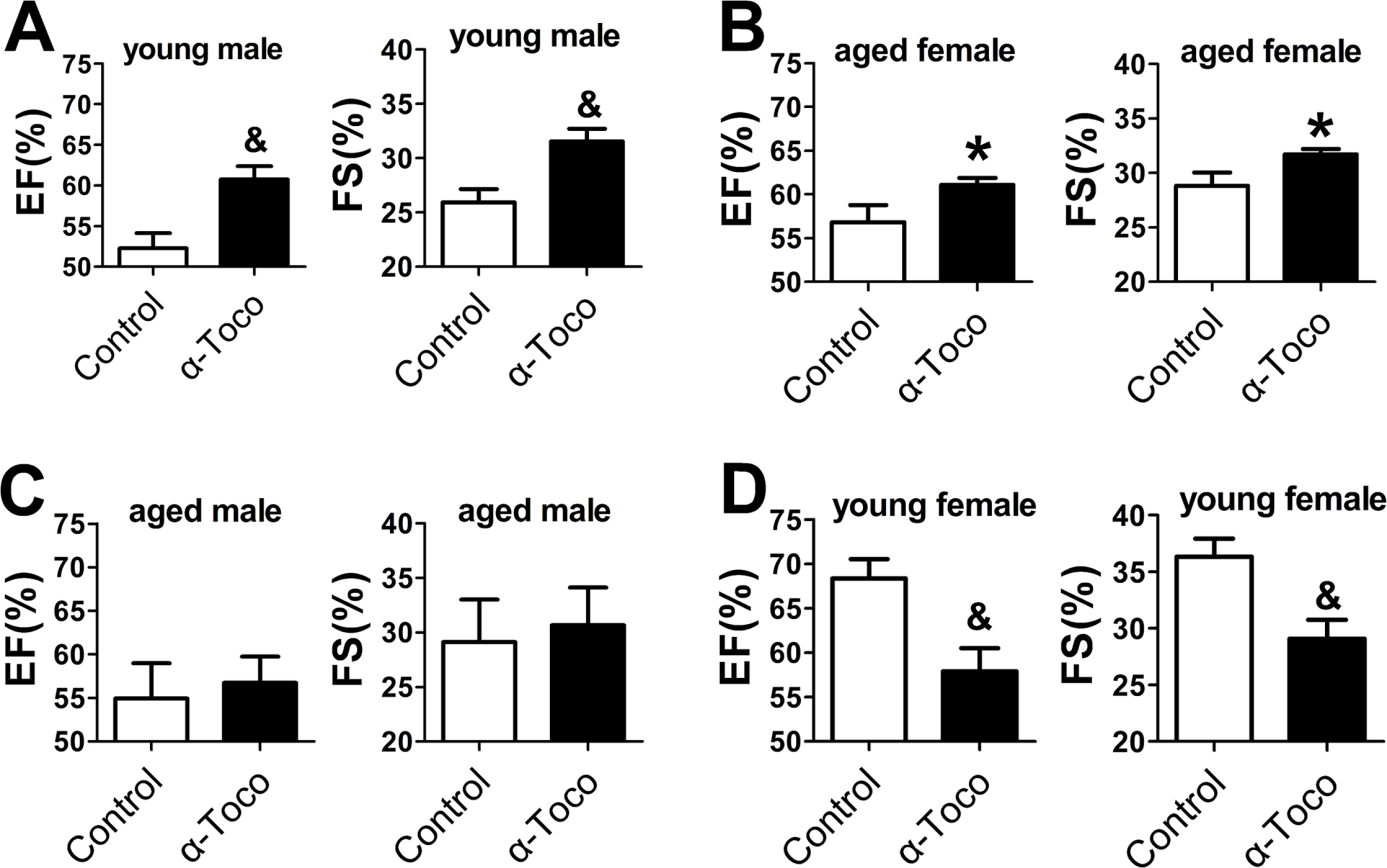
The effect of α-toco on the LV ejection fraction (EF; shown by %) and the LV fractional shortening (FS; shown by %). Comparisons were made with the values of the control group. **A and B** α-toco significantly improved the EF and FS in young male and aged female mice. **C** There was no difference in the EF and FS between the α-toco and the control groups of the aged male mice. **D** α-toco decreased the EF and FS in young female mice. n = 10, *P<0.05, & P<0.01.

We examined the effect of α-toco on cardiac function using echocardiography 24 h after MI/R (Fig 1). As shown in Fig 1A, significant increases in EF (61%±5.0 vs. 52%±5.1, p<0.01) and FS (32%±3.6 vs. 26%±3.3, p<0.01) were detected compared with the control group in the young male mice. In the aged female mice pretreated with α-toco, the EF (61%±1.9 vs. 56%±4.9, p<0.05) and FS (32%±1.2 vs. 28%±3.0, p<0.05) were significantly higher than the values in the control group (Fig 1B). α-toco showed no effect in increasing EF (59%±1.1 vs. 55%±1.4, p>0.05) and FS (31%±7.7 vs. 28%±9.4, p>0.05) in aged male mice compared with the control group (Fig 1C). α-toco pretreatment even decreased EF (57%±7.4 vs. 68%±4.9, p<0.01) and FS (29%±4.7 vs. 36%±3.4, p<0.01) in young female mice compared with the control group. In each age/gender group, there were no differences in IVSd, LVIDd, LVISd between control and α-toco group (Table 1). The basal morphological data of the mice were shown in Table 2

**Table 1:** The values of IVSd, LVIDd and LVISd in each group.

Group	Number	Control	α-toco
**IVSd(mm)**			
Young male	10	0.85±0.07	0.75±0.10
Aged female	10	1.04±0.05	1.07±0.08
Aged male	10	1.0±0.17	1.04±0.07
Young female	10	0.88±0.12	0.96±0.05
**LVIDd(mm)**			
Young male	10	3.10±0.12	3.0±0.34
Aged female	10	3.2±0.31	3.14±0.11
Aged male	10	3.58±0.59	3.24±0.8
Young female	10	2.38±0.43	2.48±0.29
**LVISd(mm)**			
Young male	10	2.29±0.02	2.14±0.18
Aged female	10	2.3±0.32	2.12±0.84
Aged male	10	2.56±0.70	2.3±0.73
Young female	10	1.54±0.34	1.77±0.23

In each group divided by age and gender, there were no differences in IVSd,LVIDd, LVISd between control and a-toco group

**Table 2:** The baseline data of four different age-/sex-mice.

Group	Number		Age (months)		Body weight (g)	
	Control	α-toco	Control	α-toco	Control	α-toco
**Young male**	25	25	3	3	25.1±0.8	24.6±1.3
**Aged female**	25	25	12	12	27.9±1.7	28.1±1.3
**Aged male**	25	25	12	12	33.0±2.2	32.1±2.1
**Young female**	25	25	3	3	21.5±0.7	21.0±1.0

In each group divided by age and gender, there were no differences in body weight between control and a-toco group

### Effects of α-toco on myocardial infarct size after MI/R

**Fig 2:**
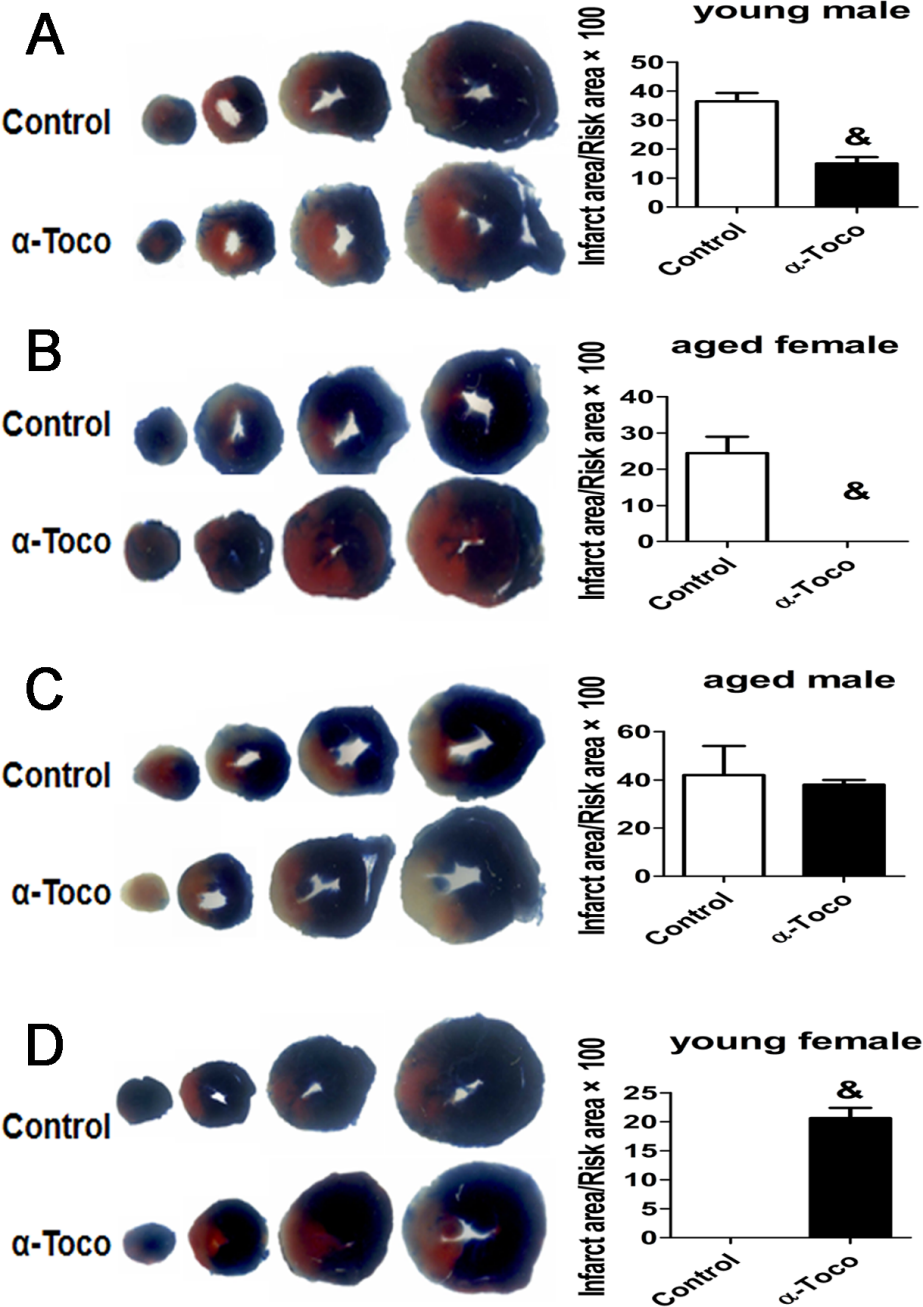
The effect of α-toco on myocardial infarct size was measured using the TTC procedure. **A and B** The myocardial infarct size of the α-toco group was significantly reduced compared with that in the control group in the young male and aged female mice. **C** There was no difference in myocardial infarct size between the α-toco and the control groups of the aged male mice. **D** α-toco increased the myocardial infarct size in young female mice. n = 5, & P<0.01.

The myocardial infarct size was determined using the TTC procedure (shown in Fig 2). Compared with the control group, the administration of α-toco in young male mice significantly decreased the myocardial infarct size (15.3%±3.7 vs. 33%±7.6, p<0.01, as shown in Fig 2A). Similarly, aged female mice pretreated with α-toco showed smaller myocardial infarcts than those of the control group (0 vs. 23.5%±4.6, p<0.01, as shown in Fig 2B). In aged male mice, there was no significant difference in the myocardial infarct size between the control and α-toco group (38.0%±3.5 vs. 40.4%±9.1, p>0.05, as shown in Fig 2C). Comparing to the control group, pretreatment with α-toco increased the myocardial infarct size in young female mice (20.6%±2.4 vs. 0, p<0.01, as shown in Fig 2D).

### Effects of α-toco on cardiomyocyte apoptosis after MI/R

**Fig 3:**
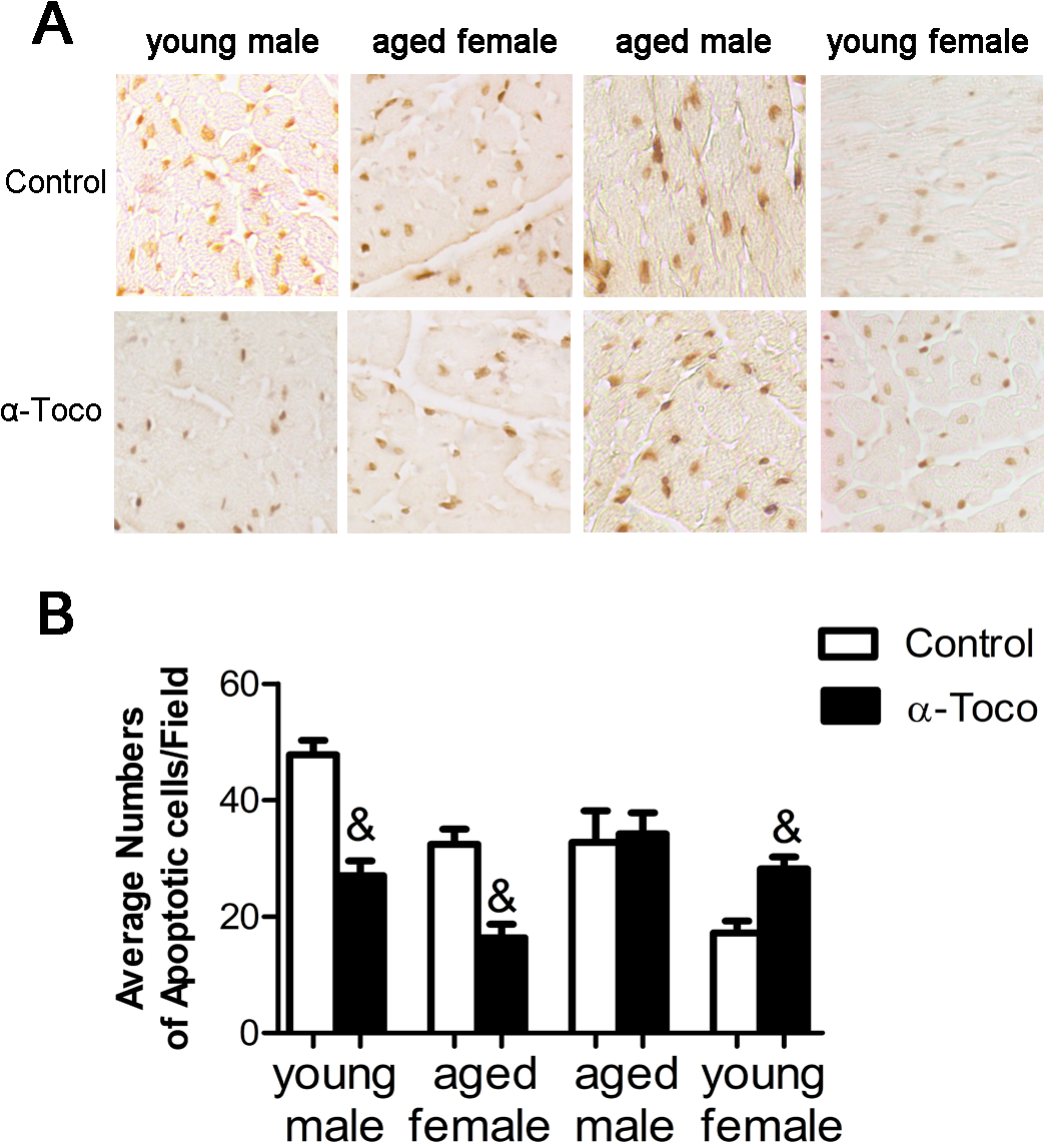
Effects of α-toco on cardiomyocyte apoptosis detected by the TUNEL procedure 24h after MI/R. Data are presented as Mean ± SD, the average number of apoptotic cells /field was compared in each group of mice. **A** Heart tissues from mice after 24h of MI/R were examined by TUNEL staining (dark brown nuclei are positive). **B** Bar graph showing average number of TUNEL-positive staining cells. n = 6, & P<0.01.

As shown in Fig 3, cardiomyocyte apoptosis was measured using the TUNEL method 24 hours after MI/R. In young male and aged female mice, the number of apoptotic cells was substantially smaller in the hearts pretreated with α-toco (27% ± 5.7% vs. 47.8% ± 5.5%, p<0.01 and 16.4% ± 5.2% vs. 32.4% ± 5.9%, p<0.01, respectively). However, α-toco did not reduce the number of apoptotic cardiomyocytes in the aged male mice (34.2% ± 8.1% vs. 32.8% ± 1.2%, p>0.05) and even increased the number of apoptotic cardiomyocytes in young female mice (28.4% ± 5.7% vs. 17.4% ± 5.6%, p<0.01).

### Effects of α-toco on cardiac proteins after MI/R

**Fig 4:**
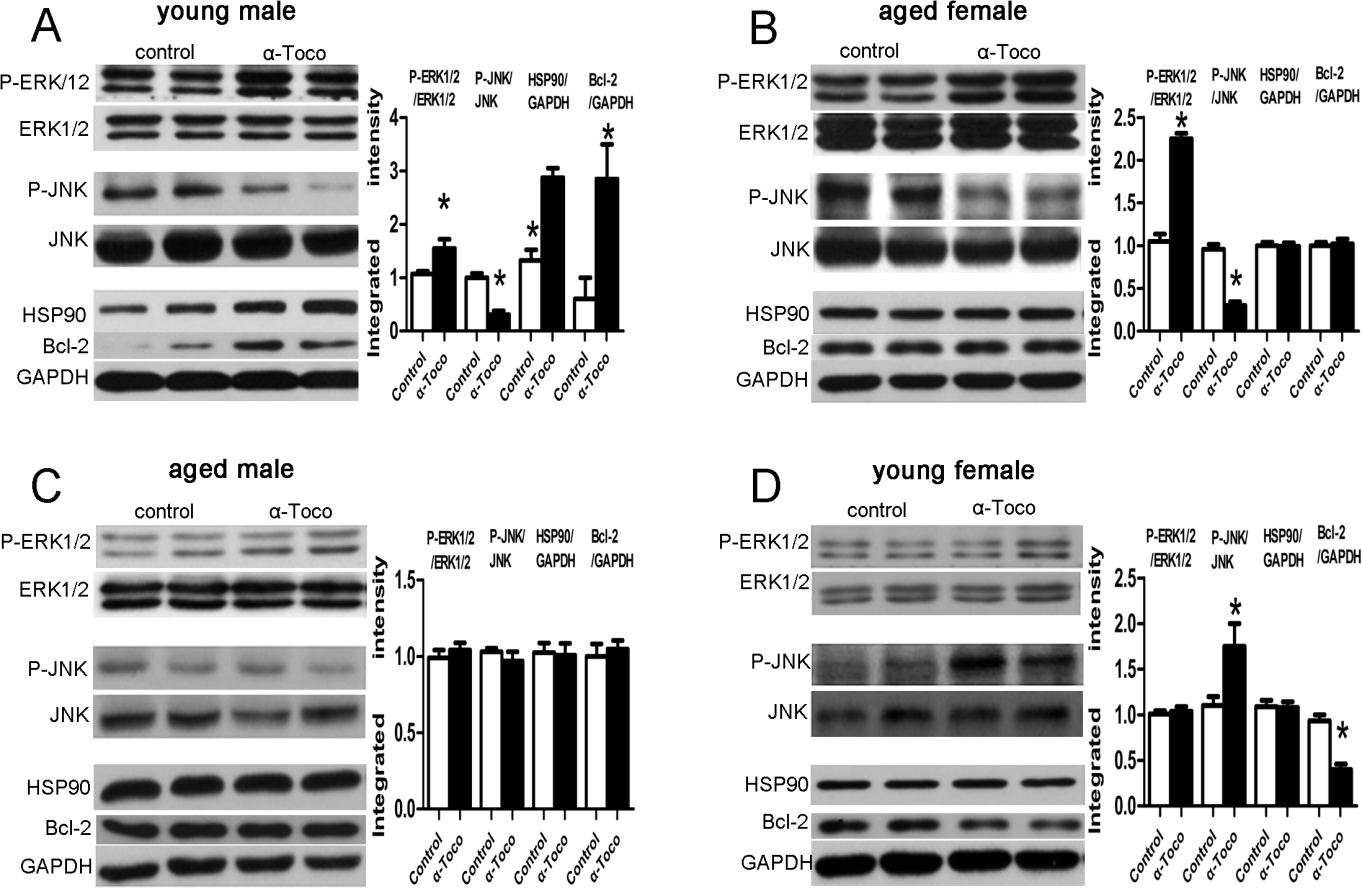
Effects of α-toco on the phosphorylation of ERK1/2 and JNK and the expression of HSP90 and Bcl-2 24 h after MI/R. Data are presented as Mean ± SD, the values were compared with those of the control in each group. **A** α-toco increased the expression of HSP90 and Bcl-2 and the phosphorylation of ERK1/2 and reduced the phosphorylation of JNK in young male mice. **B** α-toco increased the phosphorylation of ERK1/2 and reduced the phosphorylation of JNK in aged female mice. **C** No significant differences in the phosphorylation of ERK1/2 and JNK or in the expression of HSP90 and Bcl-2 were detected for aged male mice. **D** α-toco increased the phosphorylation of JNK and reduced the expression of Bcl-2 in young female mice. However, no changes were detected for ERK1/2 or HSP90. n = 6, *P<0.05.

ERK1/2, phosphorylated ERK1/2, JNK, phosphorylated JNK, and expression of HSP90 and Bcl-2 were examined 24 h after the MI/R procedure. Pretreatment with α-toco increased the phosphorylation of ERK1/2 and reduced the phosphorylation of JNK in young male and aged female mice (Fig 4A and 4B). Fig 4A also showed that pretreatment with α-toco could increase expression of HSP90 and Bcl-2 in young male mice. In aged male mice, there was no significant difference in the phosphorylation of ERK1/2 or JNK, as well as the expression of HSP90 and Bcl-2 between the control and α-toco groups (Fig 4C). α-toco could increase the phosphorylation of JNK and reduce the expression of Bcl-2 in young female mice; however, no difference was observed for the phosphorylation of ERK1/2 or for HSP90 (Fig 4D).

## Discussion

In the present study, we investigated the cardioprotective effect of VitE **(**α-toco) pretreatment in mice of different genders/ages. The results suggested that the benefit of VitE may be highly correlated with gender and age. α-toco (100 IU/ kg, gavage) can effectively reduce the hazard induced by MI/R injury in young male and old female mice, as shown by the improved left ventricular function and reduced myocardial infarct size and cardiomyocyte apoptosis. For the old male mice, pretreatment with α-toco did not effectively reduce the hazard induced by MI/R injury. For the young female mice, however, the additional supplementation of α-toco exacerbated MI/R injury, as shown by the reduced left ventricular function and increased myocardial infarct size and cardiomyocyte apoptosis.

It has been demonstrated that anti-apoptotic proteins ERK1/2, Bcl-2, HSP90 and pro-apoptotic protein JNK play roles in cardioprotective effect [20–22]. Activation of ERK1/2, JNK and expression of HSP90 could be regulated by α-toco (vitamin E) [23,24]. A previous study also demonstrated that supplementation with α-toco phosphate could decreased MI/R injury by enhancing ERK1/2 and Bcl-2, and decreasing JNK activity [25]. So we speculated that α-toco could exert cardioprotective effect via regulation of ERK1/2, JNK, Bcl-2, and HSP90 pathways. In this study, we showed that pretreatment with α-toco could converted the MAP kinase-induced death signal into a survival signal by enhancing ERK1/2 activity and reducing JNK activity in young male and aged female mice. Moreover, increased expression of HSP90 and Bcl-2 were detected in the young male mice. However, no differences were found in the phosphorylation of ERK1/2, JNK kinase and expression of HSP90 or Bcl-2 for the old male mice. For the young female mice, additional supplementation of α-toco increased the activity of pro-apoptotic JNK and reduced the expression of Bcl-2. Taken together, our findings indicated that the MAPK, HSP90 and Bcl-2 signaling pathways are involved in the cardioprotective effect of α-toco in the MI/R models of mice. It is possible that α-toco exert its anti-apoptotic or pro-apoptotic effects (according to age and gender) through these pathways. The change of those cardiac proteins were consistent with the results of left ventricular function, myocardial infarct size and cardiomyocyte apoptosis.

Unlike of water-soluble vitamins,fat-soluble vitamin E can accumulated in body [26], leading to hypervitaminosis E and unwanted side effects. It has been reported that the serum and visceral levels of α-toco decreased during aging in rats [11], and the α-toco level of the normal female animals was also higher than that of males ones [17]. These data showed that age and gender may affect the excretion and metabolism of α-toco, indicating that effective dosage and (or) interval of α-toco supplementation might be different for the cardiac protective effect in animals. And thus we have the following hypothesis: for young male and aged female mice, the normal α-toco level was not sufficient to decrease MI/R injury, and supplementation of α-toco (100 IU/kg, 21 days) increased α-toco level to the effective drug concentration to decrease MI/R injury. But for aged male mice, the physiological level of α-toco is too low and supplementation of α-toco (100 IU/kg, 21 days) did not successfully increased the α-toco level to the effective concentration to decrease MI/R injury. For young female mice, the normal α-toco level is sufficient to exert cardiac protective effect against MI/R injury, and additional supplementation of α-toco (100 IU/kg, 21 days) resulted in an excessive level of VitE, which triggered an unwanted side effect. It has been reported that VitE plays an important role in regulating the levels of sex hormones [10, 11], which affect cardiovascular function and myocardial remodeling [12]. Our data showed that age and gender should be considered when using VitE supplementation for the prevention of CV diseases in animal studies and clinical trials.

## Limitations

Firstly, we assumed that the basal cardiac functions in each group before α-toco administration and before MI/R were same, so these values were not measured. Secondly, although anesthesia were used during echocardiography, we assumed that this dosage of anesthesia would affect the cardiac function in the same manner in each group of mice. Thirdly, the concentration of VitE before α-toco administration and before MI/R procedure were not measured. Besides, myocardial infarct can affect both systolic and diastolic function, and here we didn’t measure the diastolic function in mice by echocardiography. And at last, the survival rate of the mice was not recorded. These listed limitations should be improved in future studies.

## Conclusion

In the present study, we showed that the long-term use of VitE can reduce MI/R injury by increasing anti-apopotic cardiac proteins and reducing pro-apopotic cardiac proteins to inhibit cardiomyocyte apoptosis in young male mice and old female mice but not in aged male mice. VitE enhanced MI/R injury via increasing pro-apopotic cardiac proteins and reducing anti-apopototic cardiac proteins to increase cardiomyocyte apoptosis in young female mice. Thus, the efficacy of VitE in cardiac protection is associated with age and gender. Our study suggests that age and gender should be considered when designing comparative experiments in animal studies and clinical trials. Age and gender should be considered when Vitamin E supplementation is used in the prevention of CV diseases.
